# Identifying Downregulation of Autophagy Markers in Kawasaki Disease

**DOI:** 10.3390/children7100166

**Published:** 2020-10-04

**Authors:** Fu-Chen Huang, Ying-Hsien Huang, Ho-Chang Kuo, Sung-Chou Li

**Affiliations:** 1 Department of Pediatrics, Kaohsiung Chang Gung Memorial Hospital and Chang Gung University College of Medicine, Kaohsiung 833, Taiwan; yhhuang123@yahoo.com.tw (Y.-H.H.); erickuo@yahoo.com.tw (H.-C.K.); 2 Kawasaki Disease Center, Kaohsiung Chang Gung Memorial Hospital, Kaohsiung 833, Taiwan; 3 Genomics and Proteomics Core Laboratory, Department of Medical Research, Kaohsiung Chang Gung Memorial Hospital and Chang Gung University College of Medicine, Kaohsiung 833, Taiwan; raymon.pinus@gmail.com

**Keywords:** autophagy, Kawasaki disease, children, leukocytes, coronary artery lesion

## Abstract

Kawasaki disease (KD) is the most common cause of heart disease acquired in childhood. Even if treated with high-dose intravenous immunoglobulin G (IVIG) at the early stage; children are still at risk of developing coronary artery lesions. Accumulating evidence suggests that autophagy is enhanced in various heart diseases. Evaluating the pathogenic role of autophagy in KD and coronary artery lesions (CAL) may aid in identifying a potential therapeutic target for the treatment or prevention of the disease. Blood samples were obtained from 20 children with KD at the onset of disease and 21 days after IVIG therapy. Twenty children with other causes of febrile disease and 20 healthy children were included as controls. Total RNA was extracted from white blood cells; and autophagy-related gene mRNA expression levels were measured using real-time polymerase chain reaction. The patients with KD had downregulated levels of *LC3B* mRNA (0.50 ± 0.06 vs. 1.67 ± 0.15; *p* < 0.001), *BECN* *1* mRNA (0.70 ± 0.08 vs. 1.43 ± 0.23; *p* < 0.05), and *ATG16L1* mRNA (0.28 ± 0.04 vs. 0.96 ± 0.16; *p* < 0.01) compared to the febrile control group. The values of these parameters all increased significantly 21 days after the IVIG therapy as follows: *LC3B* mRNA (1.77 ± 0.29 vs. 0.50 ± 0.06; *p* < 0.001), *BECN* *1* mRNA (1.67 ± 0.36 vs. 0.70 ± 0.08; *p* < 0.05), and *ATG16L1* mRNA (2.96 ± 0.43 vs. 0.28 ± 0.04; *p* < 0.001), while the level of *ATG16L1* mRNA persists low in KD patients with CAL. Our results showed the autophagy-related genes expressions in KD and their change after IVIG administration. This suggests that autophagy may have a protective effect on KD.

## 1. Introduction

Kawasaki disease (KD) is the most common cause of heart disease in childhood in the developed nations [1]. It is also known as mucocutaneous lymph node syndrome [2] and typically presents with fever for more than 5 days in children. Without treatment, a subset of patients (24%) tend to develop coronary artery lesions (CAL) with aneurysms in 8% [3]; these patients are inclined to complications of the heart and sudden death [4] and the mortality rate for this patient group is approximately 1–2% [5]. Administration of high-dose intravenous immunoglobulin (IVIG) plus aspirin early in the course of KD reduces the prevalence of coronary artery abnormalities from 18 to 4% [6]. Despite this success, children with persistent or recurrent fever are at an increased risk of developing CAL [7]. Additional therapies administered to these patients include retreatment with IVIG, use of immunomodulatory agents, such as cyclosporine, cyclophosphamide, and infliximab, plasma exchange, and pulsed steroid therapy with methylprednisolone [4]. Identification of the pathogenesis of KD and its complications, as well as evaluation of more effective therapy, are mandatory to prevent complications of the coronary artery in the patients with KD.

Autophagy is the major intracellular degradation system that removes unnecessary or dysfunctional components and serves as a dynamic recycling system for cellular renovation and homeostasis. Autophagy at low basal levels maintains the cardiac function and morphology of the normal heart. Autophagy is activated in response to the cellular stress that develops in almost all forms of heart disease [8]. However, studies on the functional role of autophagy in the diseased heart have conflicting results. In some cases, enhanced autophagic responses provide protective effects on cardiovascular diseases, while in others, it promotes deterioration in disease. There is increasing evidence that autophagy is enhanced in various cardiovascular diseases, including cardiac hypertrophy [9], cardiomyopathy [10], valvular and hypertensive heart disease [11], heart failure [12], and during myocardial ischemia and reperfusion [13]. Taken together, these studies support the current role of autophagy in cardiovascular diseases though the results are conflicting [8]. Thus, evaluating the pathogenic role of autophagy in KD and CAL may aid in identifying a potential therapeutic target for the treatment or prevention of the disease.

Therefore, this study aimed to investigate the expression of autophagy markers, including Beclin 1, LC3, and autophagy related 16 like 1 (ATG16L1), in the peripheral leukocytes in children with KD.

## 2. Patients and Methods

### 2.1. Patients

This study was approved by the Institutional Review Board of the Chang Gung Memorial Hospital (no. 201601024B0), and informed consent was obtained from either the parents or guardians of the children. All children diagnosed with KD were treated with IVIG (2 gm/kg) infusion slowly. Blood samples were obtained at the onset (before IVIG treatment; KD1) and 21 days after IVIG treatment (KD3). Patients who did not fulfill the diagnostic criteria of KD were excluded. We measured the internal lumen diameters of the coronary artery using two-dimensional echocardiography. CAL were defined as when the internal diameters of the coronary artery were greater than 3 mm (or 4 mm if the subject was aged more than 5 years) or when the internal diameter of a segment was at least 1.5 times that of the adjacent segment, and by a clearly irregular luminal contour pursuant to the guidelines of the Japanese Ministry of Health [14]. Age-matched patients who had fever caused by other etiologies, including herpangina, acute bronchiolitis, acute pharyngotonsillitis and acute sinusitis, and did not have a history of KD constituted the febrile control group. From the outpatient clinics, we enrolled another 20 healthy children (without any history of KD) who tested negative for an allergen test and volunteered to participate in our study as the healthy control group. The demographic and clinical characteristics of these children are shown in the Tables.

### 2.2. Real-Time PCR

We collected 3–5 mL whole blood samples from all of the participants and submitted them to white blood cell (WBC) enrichment. Total RNA was extracted from the leukocytes of patients with KD and from the leukocytes of the febrile and healthy control groups as previously reported [15]. Reverse transcription step was using TaKaRa PrimeScript™ RT reagent Kit (TaKaRaCat #RR037A; Takara Bio Inc., Shiga, Japan) in 20-μL reactions and a final concentration of 1 μg total RNA, 5x PrimeScript buffer, 50 μM Oligo dT primer, 100 μM Random 6 mers, PrimeScript RT Enzyme Mix1 and the total volume was transferred at 20 μL per tube with RNase Free dH_2_O. Reverse transcription was carried out in a ABI 2720 Thermal cycler (Applied Biosystems, CA, USA), programmed to run the reverse transcription for 15 min at 37 °C, and inactivation the reverse transcriptase for 5 s at 85 °C and finally incubated the cDNA products at 4 °C. Before the PCR step, the cDNA products were stored at −20 °C.

The mRNA levels of autophagy markers, *LC3B, BECN 1, and ATG16L1*, were measured by using Real-Time RT–PCR twostep multiplex technique in an ABI PRISM^TM^ 7700 (Applied Biosystems, CA, USA). Probes and primers were obtained from Applied Biosystems. Real time PCR experiments were carried out by ABI 7500 Fast Real-Time PCR System (Applied Biosystems). The reagent used the Fast SYBR Green Master Mix (Thermo Fisher #4385612, Applied Biosystems, CA, USA) and followed the manufacturer’s instructions. The reaction mix contained 5 μL of sample mixed with 15 μL of PCR cocktail (200 nM for each primer). The reaction for each well was carried out as follows: 95 °C for 20 s, followed by 95 °C for 3 s and 60 °C for 30 s and repeated for 40 cycles. Dissociation stage was carried out as follows, 95 °C for 15 s, 60 °C for 60 s, 95 °C for 15 s and 60 °C for 15 s. The ABI7500 software (SDS V2.3) was used to obtain raw fluorescence data (Rn and DRn) for analysis. Many aspects of MIQE guidelines were taken into consideration for methods and analysis [1]. The primers used for amplification were listed in supplemental table (Table S1). The number of transcripts was calculated using the comparative threshold cycle (2^ΔΔCt^) formula, with the endogenous *GAPDH* expression as the internal control.

### 2.3. Statistical Analysis

Statistical analyses were performed using the paired Student’s *t*-test or Mann–Whitney U test for comparison of two parametric or nonparametric variables and Kruskal–Wallis one-way analysis of variance for the comparison of nonparametric three or more variables (GraphPad Prism 8; GraphPad software, San Diego, CA, USA). A *p*-value of <0.05 was considered statistically significant.

## 3. Results

### 3.1. Demographic Data

In all, 20 children with KD (aged 1.40 ± 0.69 years; 6 males and 14 females) were included in this study. Twenty patients with an acute febrile infectious disease (aged 1.49 ± 0.78 years; 12 males and 8 females) and 20 healthy children (aged 1.47 ± 0.61 years; 14 males and 6 females) were enrolled as the controls. There was no significant difference in age and sex between the children with KD and those in both the control groups (Table 1, Table 2, Table 3, Table 4 and Table 5).

### 3.2. Differential Counts of Leukocytes and Monocytes among the Control Groups and the Children with KD before and after Treatment

There was no significant difference in the differential counts of neutrophils (30.75 ± 11.1% vs. 39.05 ± 22.12%, *p* = 0.287) and monocytes (6.18 ± 2.29% vs. 8.15 ± 4.31%, *p* = 0.202) between the healthy and febrile control groups (Table 1). However, we observed a significant increase in the differential counts of leukocytes (59.79 ± 10.4%, *p* < 0.05) in children with KD before treatment (Table 2 and Table 3) and a decrease in the differential counts of monocytes (4.90 ± 1.13%, *p* < 0.05) in children with KD after treatment (Table 4 and Table 5) compared to both the control groups. Although the differential counts of lymphocytes were different between the KD1 and control groups, the total number of lymphocytes was not significantly different.

### 3.3. Analyses of Autophagy Markers mRNA in the Peripheral White Blood Cells of Kawasaki Disease (KD) Patients before and after IVIG Treatment

**Figure 1:**
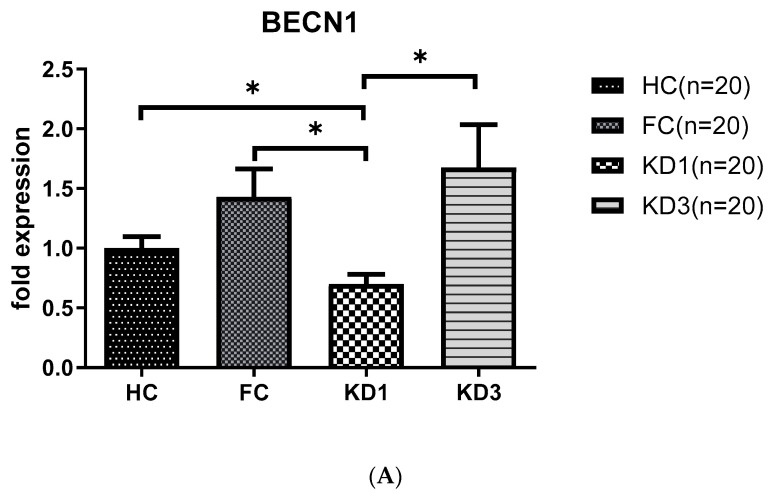

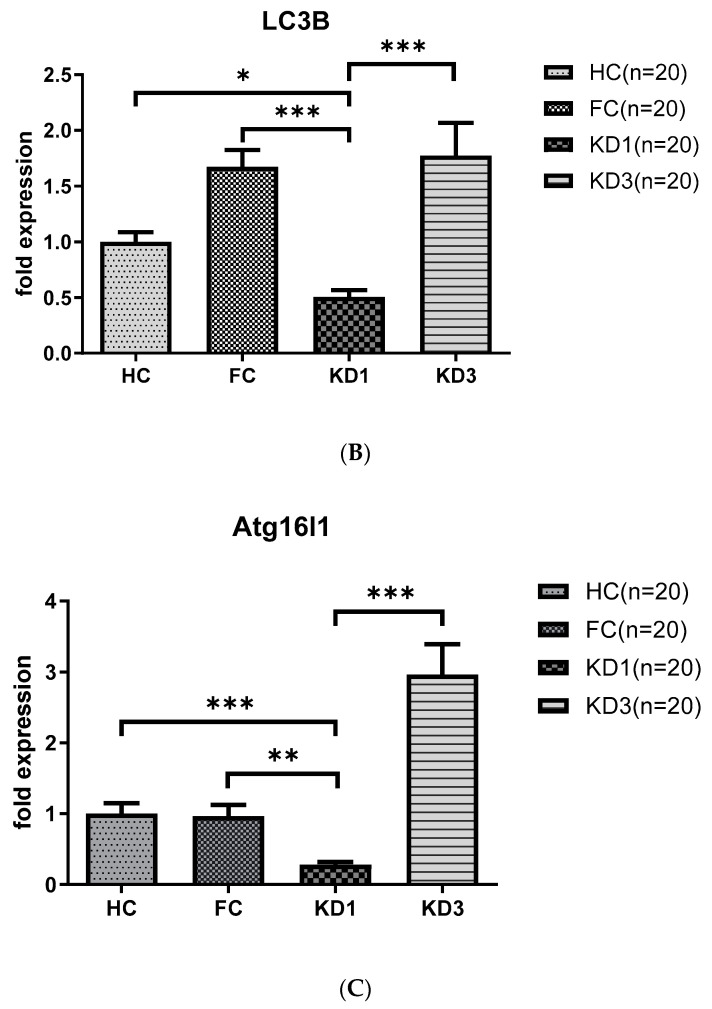
Analyses of mRNA autophagy markers in the peripheral white blood cells of Kawasaki disease (KD) patients before and after IVIG treatment. Total RNA from the peripheral white blood cells of healthy controls (HC, *n* = 20), febrile controls (FC, *n* = 20) and KD patients before (KD1, *n* = 20) and after (KD3, *n* = 20) IVIG treatment were isolated using a total RNA purification kit. Reverse transcription was performed using a high-performance reverse transcriptase system as described in the Methods section. The levels of *BECN 1* (**A**), *LC3B* (**B**) and *ATG16L1* (**C**) mRNA was measured by a real-time quantitative polymerase chain reaction (RT-qPCR). The expression levels were normalized to those of GAPDH. The comparisons among these groups were analyzed. Data are expressed as mean ± standard error. * *p* < 0.05, ** *p* < 0.01, *** *p* < 0.001 (by Kruskal–Wallis one-way analysis of variance).

As shown in Figure 1, we observed that patients with KD before treatment (KD1) had downregulated levels of *LC3B* (0.50 ± 0.06 vs. 1.67 ± 0.15, *p* < 0.001), *BECN 1* (0.70 ± 0.08 vs. 1.43 ± 0.23, *p* < 0.05) and *ATG16L1* (0.28 ± 0.04 vs. 0.96 ± 0.16, *p* < 0.01) mRNA compared to the febrile control and healthy control groups. The produced mRNA of all these markers increased significantly 21 days after IVIG therapy (KD3)(*LC3B*, 1.77 ± 0.29 vs. 0.50 ± 0.06, *p* < 0.001; *BECN 1*, 1.67 ± 0.36 vs. 0.70 ± 0.08, *p* < 0.05; and *ATG16L1*, 2.96 ± 0.43 vs. 0.28 ± 0.04, *p* < 0.001) compared to KD1.

### 3.4. The Lower Levels of ATG16L1 mRNA Persist in KD Patients with CAL Compared to Those without CAL 21 Days after IVIG Therapy

**Figure 2:**
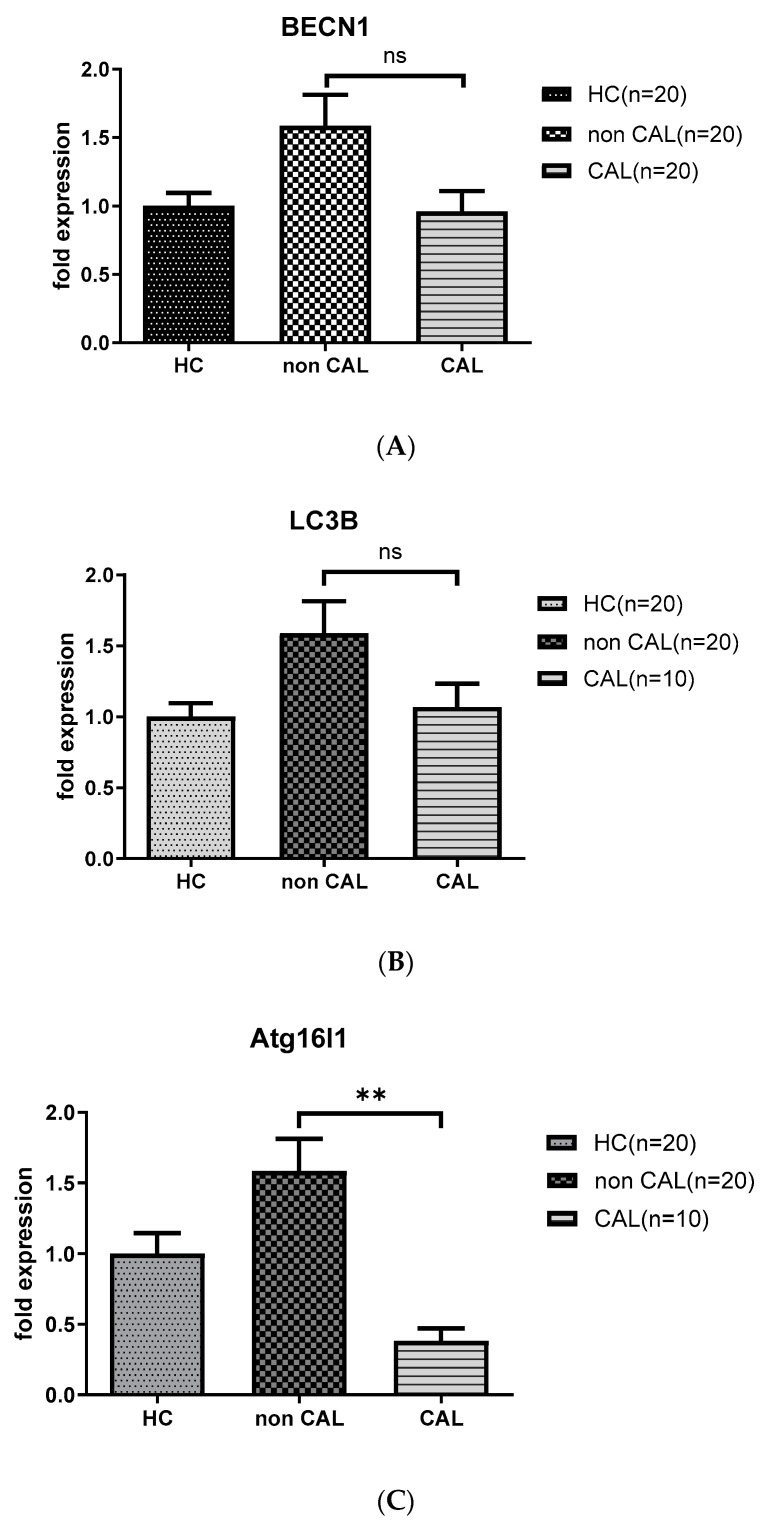
Analyses of mRNA autophagy markers in the peripheral white blood cells of Kawasaki disease (KD) patients without and with coronary artery lesions. Total RNA from the peripheral white blood cells of healthy controls (HC, *n* = 20) and KD patients without (non CAL, *n* = 20) and with coronary artery lesions (CAL, *n* = 10) 21 days after (KD3) IVIG treatment was isolated using a total RNA purification kit. Reverse transcription was performed using a high-performance reverse transcriptase system, as described in the Methods section. The levels of *BECN 1* (**A**), *LC3B* (**B**) and *ATG16L1* (**C**) mRNA was measured by a real-time quantitative polymerase chain reaction (RT-qPCR). The expression levels were normalized to those of *GAPDH*. The comparisons between the two groups of KD patients were analyzed. Data are expressed as mean ± standard error. ** *p* < 0.01 (by Mann–Whitney U test).

As shown in Figure 2, KD patients with CAL (CAL; *n* = 10) had persistent downregulated levels of *ATG16L1* mRNA (0.38 ± 0.04 vs. 2.85 ± 0.46, *p* < 0.01) compared to the age-matched KD patients without CAL (non CAL; *n* = 20) 21 days after IVIG therapy; while the levels of *BECN 1* and *LC3B* mRNA not significantly different between the aforementioned groups.

## 4. Discussion

In the normal heart, basal levels of autophagy is observed, and autophagy is characterized by maintaining a homeostatic status of heart. However, the functional studies on autophagy in the diseased heart have reported conflicting results [8,10,11,12,13,16,17,18]. In some cases, increased autophagy is beneficial and promotes functional recovery, and the myocardium may be salvaged after ischemia/reperfusion injury [8,13,17,18,19]; however, in other cases, autophagy is associated with cell death and promotion of heart failure [8,10,11,12]. Some studies suggest the role of autophagy proteins, Beclin 1 and LC3B, in the cardioprotection of the ischemic heart. In the current study, we observed that patients with KD had lower mRNA levels of autophagy markers, including LC3, Beclin 1, and ATG16L1 compared to the febrile control group. Additionally, the expression of these markers significantly increased 21 days after IVIG therapy.

Although the etiology of KD remains unknown, it is believed a combination of microbial infection, immune response or genetic susceptibility contribute to the development of KD. Both clinical and epidemiological characteristics of KD strongly suggest the involvement of infectious agents in the onset of KD [20]. Most studies report that an infectious agent triggers the illness [21,22,23,24], although the infectious agent remains unidentified at present. Heterogeneous infectious etiologies may account for the development of KD in different countries and during different seasons. In our study, neutrophils, which were predominantly noted in the patients with KD before treatment, may indicate the triggering role of bacterial infection in KD. The lower levels of autophagy in children KD in our study suggests that unknown microorganisms may play an important role in triggering KD [25,26], with poor autophagic clearance of these pathogens. The expression of *ATG16L1* T300A variant decreases selective autophagy, leading to altered cytokine signaling and decreased antibacterial defense [27]. Neutrophils are the most abundant leukocytes and play a central role in the primary mechanisms underlying host defense. IVIG enhances the killing and autophagy activities of neutrophils isolated from either immunocompetent [28] or immunocompromised [29] patients exposed to multidrug-resistant bacteria. We revealed that the mRNA expression of autophagy markers was lower in the leukocytes of patients with KD who predominantly had neutrophils (Table 2 and Table 4). This can explain the therapeutic effect of IVIG on patients with KD [30] and the higher incidence of CAL in patients with IVIG-resistant KD [31]. Neutrophil-mediated damage of endothelial cells [32] contributes towards the generation of CAL in patients with KD. The infiltration of neutrophils in CAL during the acute phase of KD contributes towards the initial damage to coronary arteries, while sustained activity of neutrophils [33] may be involved in the pathogenesis of KD vasculitis [34]. Effective elimination of neutrophils is a resolution of detrimental inflammatory response. Most of the current data suggest that autophagy assists death of the neutrophils [35]. The absence of autophagy may contribute to sustained activity of neutrophils, leading to the injury of endothelial cells, while IVIG enhances neutrophil autophagy [28] and death of neutrophils and prevents the development of CAL in patients with KD.

The increase in autophagy in macrophages after ingesting pathogens contributes towards pathogen defense mechanisms, while dysregulated autophagy may allow pathogen survival in macrophages and stimulation of inflammatory responses. Macrophage autophagy limits acute toxic liver injury in mice through downregulation of interleukin-1β expression [36]. Several clinical studies on KD suggest that serum levels of IL-1β or IL-1β, gene expression may play an essential role in KD [37,38] and development of CAL [39]. Decreased expression of the autophagy protein, ATG16L1, enhances endotoxin-induced IL-1β production [40]. *ATG16L1* polymorphisms together with the excessive production of IL-1β and IL-6 in human peripheral mononuclear cells [41] may account for the chronic inflammatory process in Crohn’s disease. It is compatible with our observation that the downregulated levels of *ATG16L1* mRNA persists in KD children with CAL even after IVIG therapy (Figure 2) and may provide a mechanistic explanation for the involvement of this protein in the pathogenesis of CAL in children with KD. The persistent downregulated *ATG16L1* mRNA level in KD patients may contribute towards the marked chronic inflammation in arteritis. The levels of *ATG16L1* mRNA may be used as an indicator for CAL development in children with KD, and *ATG16L1* polymorphism or miRNA controlling the expression of ATG16L1 may play an essential role in the pathogenesis of CAL in children with KD. Moreover, our preliminary data (data not shown), based on Illumina Next generation sequencing platform and miRSeq analysis for determining miRNA expression profiles in our Lab [42], revealed a significant increase in miR142-3p and miR93-3p expression at disease onset in children with KD before administration of IVIG treatment compared to the febrile controls. Targeting of autophagy protein, ATG16L1, by miR142-3p suggests that this miRNA may play a role in intestinal inflammation and Crohn’s disease [43]. Disruption of ATG16L1-mediated autophagy by miR-93 hinders the elimination of bacteria from the epithelial cells [44]. This finding is similar to that reported in a recent study that miR-93 may participate in regulating gene expression in circulating peripheral blood mononuclear cells and contribute towards the pathogenesis of CAL in acute KD [45]. In our study, the differential counts of peripheral monocytes are decreased in KD patients 21 days after IVIG therapy, which may lead to the activation of macrophages. Macrophage autophagy downregulates IL-1β expression, leading to the repair of endothelial damage after IVIG therapy.

Additionally, autophagy has an essential regulatory function in macrophage polarization that downregulates inflammation [46]. The ingestion of dead neutrophils may also influence the macrophage phenotype [47]. In the absence of neutrophils, macrophages predominantly assume M2-like phenotypes [48]. Impaired macrophage autophagy influences macrophage polarization by increasing proinflammatory M1 and decreasing anti-inflammatory M2 polarization in obese mice [46] and leads to hepatic inflammation and liver injury. M2 macrophage polarization mediates the anti-inflammatory effects of endothelial nitric oxide signaling [49]. In Table 3 and Table 5, besides neutrophils, patients with KD presented with a decreased number of monocytes after treatment (KD3), which were supposed to be polarized into macrophages M1 or M2 in the coronary arteries. Furthermore, IVIG induces autophagy in M1 macrophages but not in M2 macrophages and reduced inflammatory cytokines in the circulation [50]. It explains in part the mechanism by which IVIG therapy benefits patients with autoimmune and inflammatory disease. However, it is difficult to differentially sort macrophages into M1 and M2 types for determining a precise count. Although the differential counts of peripheral monocytes were similar in our patient groups, it does not mean that the proportion of M1 or M2 was similar. On the other hand, a phosphopeptide P140 that directly acts on chaperone-mediated autophagy, which is hyperactivated in certain subsets of lymphocytes in lupus, can be a very promising therapeutic agent in autoinflammatory diseases [51].

This study has certain limitations. First, all of our KD patients belonged to the Taiwanese population, so our findings need to be investigated in other KD populations, and clinical samples of a second population should be considered to confirm our findings. Second, we studied the autophagy expression in peripheral WBC, but studying autophagy expression in infiltrated neutrophils of coronary arteries, monocytes or lymphocytes in circulation in early-stage KD patients could yield better results. Such research would provide important insights into the pathophysiology of autophagy in the CAL of KD, which may ultimately lead to developing novel approaches for treating the disease.

## 5. Conclusions

We observed that the downregulated mRNA levels of autophagy markers in children with KD return to normal or even exceed the normal level in the general population 21 days after IVIG therapy, and the downregulated ATG16L1 mRNA levels persists in KD children with CAL even after IVIG therapy. Thus, the data obtained support the notion that change of autophagy in both monocytes/macrophages and neutrophils contributes to elimination or development of CAL in KD children, focusing on ATG16L1; thus, raising the possibility that manipulation of autophagy can have far-reaching therapeutic benefits. However, well-controlled clinical trials are needed to determine the effects of the administration of autophagy-enhancing agents in KD children.

## Figures and Tables

**Table 1:** Demographic and Clinical Characteristics of Enrolled Patients.

	HC (*n* = 20)	FC (*n* = 20)	*p* Value
age	1.47 ± 0.61	1.49 ± 0.78	0.932
sex	♂(14)/♀(6)	♂(12)/♀(8)	0.519
WBC	9.035 ± 2.06	9.95 ± 3.47	0.371
RBC	4.81 ± 0.55	4.48 ± 0.24 *	0.033
Hemoglobin	12.32 ± 0.9	11.58 ± 0.94 *	0.047
Hematocrit	35.87 ± 2.57	35.01 ± 2.59	0.397
Platelets	314.25 ± 74.35	361.6 ± 145.1	0.243
Segment	30.75 ± 11.1	39.05 ± 22.12	0.287
Lymphocyte	58.17 ± 12.12	48.2 ± 21.08	0.110
Monocyte	6.18 ± 2.29	8.15 ± 4.31	0.202
Eosinophil	3.41 ± 2.07	1.42 ± 1.68 *	0.014
Basophil	0.49 ± 0.77	0.18 ± 0.34	0.239

All variable data are expressed as mean ± SD. FC: indicates febrile control; HC: healthy control. * *p* < 0.05.

**Table 2:** Demographic and Clinical Characteristics of Enrolled Patients.

	HC (*n* = 20)	KD1 (*n* = 20)	*p* Value
age	1.47 ± 0.61	1.40 ± 0.69	0.700
sex	♂(14)/♀(6)	♂(13)/♀(7)	0.747
WBC	9.03 ± 2.06	13.91 ± 4.98 *	0.002
RBC	4.81 ± 0.55	4.30 ± 0.60 *	0.014
Hemoglobin	12.32 ± 0.9	10.76 ± 0.94 ***	0.000
Hematocrit	35.87 ± 2.57	32.5 ± 2.50 ***	0.000
Platelets	314.25 ± 74.35	317.93 ± 94.42	0.896
Segment	30.75 ± 11.19	59.79 ± 10.40 ***	0.000
Lymphocyte	58.17 ± 12.12	30.00 ± 9.84 ***	0.000
Monocyte	6.18 ± 2.29	6.28 ± 2.77	0.899
Eosinophil	3.41 ± 2.07	2.42 ± 1.76	0.139
Basophil	0.49 ± 0.77	0.15 ± 0.26	0.078

All variable data are expressed as mean ± SD. HC: indicates healthy control and KD1: Kawasaki before intravenous immunoglobulin G (IVIG). * *p* < 0.05. *** *p* < 0.001.

**Table 3:** Demographic and Clinical Characteristics of Enrolled Patients.

	FC (*n* = 20)	KD1 (*n* = 20)	*p* Value
age	1.49 ± 0.78	1.40 ± 0.69	0.725
sex	♂(12)/♀(8)	♂(13)/♀(7)	0.747
WBC	9.95 ± 3.47	13.91 ± 4.98 *	0.038
RBC	4.48 ± 0.24	4.30 ± 0.61	0.322
Hemoglobin	11.58 ± 0.94	10.83 ± 0.96	0.062
Hematocrit	35.01 ± 2.59	32.5 ± 2.50 *	0.022
Platelets	361.60 ± 145.10	317.93 ± 94.42	0.360
Segment	39.05 ± 22.12	59.79 ± 10.4 *	0.017
Lymphocyte	48.2 ± 21.08	30.00 ± 9.84 *	0.026
Monocyte	8.15 ± 4.31	6.28 ± 2.77	0.191
Eosinophil	1.42 ± 1.68	2.42 ± 1.76	0.163
Basophil	0.18 ± 0.34	0.15 ± 0.26	0.804

All variable data are expressed as mean ± SD. FC: indicates febrile control and KD1: Kawasaki before intravenous immunoglobulin G (IVIG). * *p* < 0.05.

**Table 4:** Demographic and Clinical Characteristics of Enrolled Patients.

	HC (*n* = 20)	KD3 (*n* = 20)	*p* Value
age	1.47 ± 0.61	1.40 ± 0.69	0.75
sex	♂(14)/♀(6)	♂(13)/♀(7)	0.747
WBC	9.03 ± 2.06	8.28 ± 2.13	0.291
RBC	4.81 ± 0.55	4.53 ± 0.42	0.107
Hemoglobin	12.32 ± 0.9	11.66 ± 0.89	0.037
Hematocrit	35.87 ± 2.57	34.86 ± 1.97	0.208
Platelets	314.25 ± 74.35	337.64 ± 72.57	0.342
Segment	30.75 ± 11.19	29.43 ± 7.57	0.691
Lymphocyte	58.17 ± 12.12	61.89 ± 7.52	0.267
Monocyte	6.18 ± 2.29	4.90 ± 1.13 *	0.045
Eosinophil	3.41 ± 2.07	3.26 ± 1.15	0.797
Basophil	0.49 ± 0.77	0.32 ± 0.27	0.421

All variable data are expressed as mean ± SD. HC: indicates healthy control and KD3: Kawasaki disease after intravenous immunoglobulin G (IVIG). * *p* < 0.05.

**Table 5:** Demographic and Clinical Characteristics of Enrolled Patients.

	FC (*n* = 20)	KD3 (*n* = 20)	*p* Value
age	1.49 ± 0.78	1.40 ± 0.69	0.775
sex	♂(12)/♀(8)	♂(13)/♀(7)	0.747
WBC	9.95 ± 3.47	8.28 ± 2.13	0.140
RBC	4.48 ± 0.24	4.53 ± 0.42	0.734
Hemoglobin	11.58 ± 0.94	11.66 ± 0.89	0.828
Hematocrit	35.01 ± 2.59	34.86 ± 1.97	0.876
Platelets	361.60 ± 145.10	338.87 ± 74.89	0.594
Segment	39.05 ± 22.12	29.43 ± 7.57	0.213
Lymphocyte	48.20 ± 21.08	61.89 ± 7.52	0.075
Monocyte	8.15 ± 4.31	4.94 ± 1.09 **	0.007
Eosinophil	1.42 ± 1.68	3.26 ± 1.15 **	0.003
Basophil	0.18 ± 0.34	0.32 ± 0.27	0.245

All variable data are expressed as mean ± SD. FC: indicates febrile control and KD3: Kawasaki disease after intravenous immunoglobulin G (IVIG). ** *p* < 0.01.

## Data Availability

The datasets used and/or analyzed during the current study are available from the corresponding author on reasonable request.

## Supplementary Materials

The following are available online at https://www.mdpi.com/2227-9067/7/10/166/s1, Table S1: Primer sequences used for real time RT-PCR.
