# Supplementary material for: Identifying Downregulation of Autophagy Markers in Kawasaki Disease
Source: Children (Basel). 2020 Oct 4;7(10):166. doi: 10.3390/children7100166 (PMC7600284; doi:10.3390/children7100166)
Supplement: Supplementary file 1 [file children-07-00166-s001.pdf]

Table S1. Primer sequences used for real time RT-PCR.

Gene	Accession No.[a]	Primer sequence (5′ → 3′)	Orientation	Product size (bp)
BECN1	NM_001313998.2	AGGTTGAGAAAGGCGAGACA<br>GCTTTTGTCCACTGCTCCTC	Forward primer<br>Reverse primer	139
LC3B	NM_001085481.3	ATGCCGTCGGAGAAGACCTTCAA<br>TTAGCATTGAGCTGTAAGCGCCTTC	Forward primer<br>Reverse primer	227
ATG16L1	NM_001363742.2	TCCCAGAGTTTGAGAGTCCG<br>CGAATCTGGACTGTGGATGA	Forward primer<br>Reverse primer	137

[a] Accession number from Genbank (NCBI).
